# Diversity, Abundance, and Niche Differentiation of Ammonia-Oxidizing Prokaryotes in Mud Deposits of the Eastern China Marginal Seas

**DOI:** 10.3389/fmicb.2016.00137

**Published:** 2016-02-12

**Authors:** Shaolan Yu, Peng Yao, Jiwen Liu, Bin Zhao, Guiling Zhang, Meixun Zhao, Zhigang Yu, Xiao-Hua Zhang

**Affiliations:** ^1^Laboratory of Marine Microbiology, College of Marine Life Sciences, Ocean University of ChinaQingdao, China; ^2^Key Laboratory of Marine Chemistry Theory and Technology, Ministry of Education, Ocean University of ChinaQingdao, China; ^3^Qingdao Collaborative Innovation Center of Marine Science and Technology, Ocean University of ChinaQingdao, China; ^4^Laboratory for Marine Ecology and Environmental Science, Qingdao National Laboratory for Marine Science and TechnologyQingdao, China

**Keywords:** AOA, AOB, community structures, eastern China marginal seas, mud deposits, spatial distribution

## Abstract

The eastern China marginal seas (ECMS) are prominent examples of river-dominated ocean margins, whose most characteristic feature is the existence of isolated mud patches on sandy sediments. Ammonia-oxidizing prokaryotes play a crucial role in the nitrogen cycles of many marine environments, including marginal seas. However, few studies have attempted to address the distribution patterns of ammonia-oxidizing prokaryotes in mud deposits of these seas. The horizontal and vertical community composition and abundance of ammonia-oxidizing archaea (AOA) and bacteria (AOB) were investigated in mud deposits of the South Yellow Sea (SYS) and the East China Sea (ECS) by using *amoA* clone libraries and quantitative PCR. The diversity of AOB was comparable or higher in the mud zone of SYS and lower in ECS when compared with AOA. Vertically, surface sediments had generally higher diversity of AOA and AOB than middle and bottom layers. Diversity of AOA and AOB showed significant correlation with latitude. *Nitrosopumilus* and *Nitrosospira* lineages dominated AOA and AOB communities, respectively. Both AOA and AOB assemblages exhibited greater variations across different sites than those among various depths at one site. The abundance of bacterial *amoA* was generally higher than that of archaeal *amoA*, and both of them decreased with depth. Niche differentiation, which was affected by dissolved oxygen, salinity, ammonia, and silicate (SiO${}_{3}^{2-}$), was observed between AOA and AOB and among different groups of them. The spatial distribution of AOA and AOB was significantly correlated with δ^15^N_TN_ and SiO${}_{3}^{2-}$, and nitrate and δ^13^C, respectively. Both archaeal and bacterial *amoA* abundance correlated strongly with SiO${}_{3}^{2-}$. This study improves our understanding of spatial distribution of AOA and AOB in ecosystems featuring oceanic mud deposits.

## Introduction

Nitrification is one of the most important processes of nitrogen biogeochemical cycling in the ocean, including the oxidation of ammonia first to nitrite and subsequently to nitrate (Zehr and Kudela, 2011). The oxidation of ammonia to nitrite, the critical and rate-limiting step in nitrification, is carried out by chemolithoautotrophic ammonia-oxidizing bacteria (AOB) and ammonia-oxidizing archaea (AOA; Kowalchuk and Stephen, 2001; Könneke et al., 2005). Although both AOA and AOB are ammonia oxidizers, they are different in phylogeny and physiological characters, leading to significant variations in the relative abundance, community structure, and activity between them under different environmental conditions. For example, AOA were more abundant than AOB in the North Sea, the Sargasso Sea and the Northern South China Sea (Dang et al., 2013; Newell et al., 2013; Lipsewers et al., 2014); a positive correlation between AOA abundance and ammonia-oxidation rates has been observed. However, the abundance of AOA was decoupled from nitrification in the Central California Current (Santoro et al., 2010), and AOB was dominant in the Colne Estuary, the Cochin Estuary, and the South Atlantic Ocean (Xu et al., 2014; Li et al., 2015; Veettil et al., 2015). The relative importance of AOA and AOB in marine nitrogen cycle is therefore still under debate.

**Figure 1:**
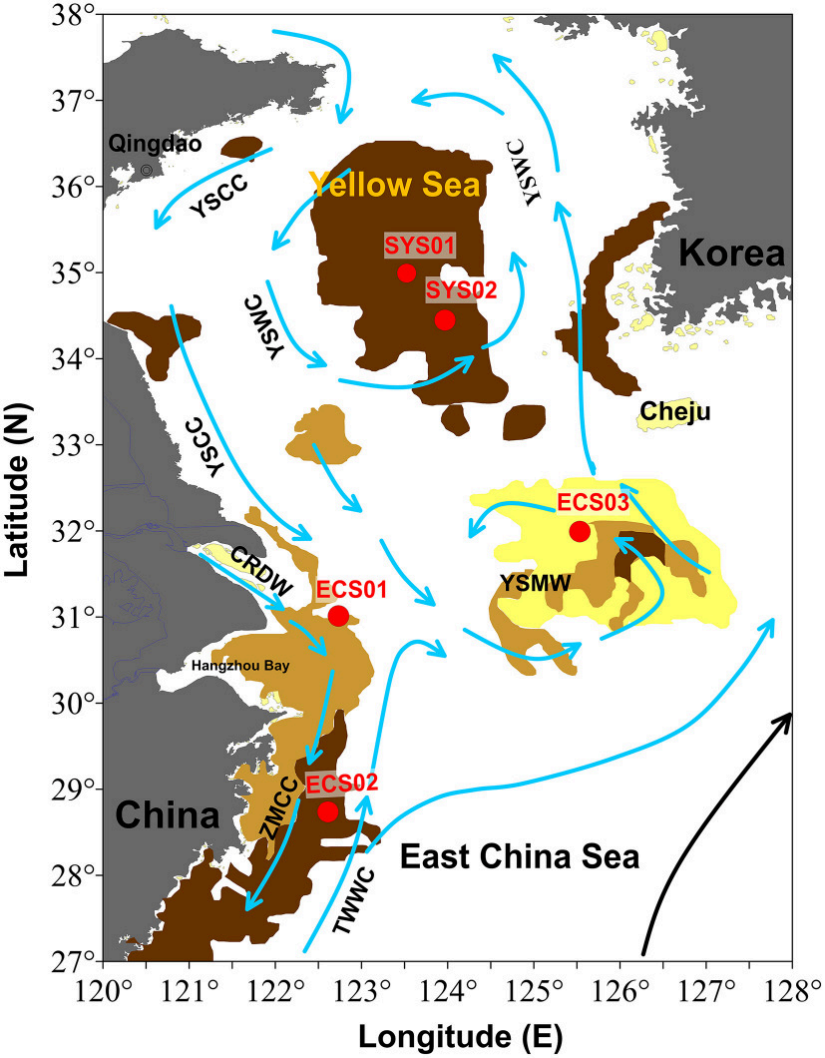
**Map showing locations of the sampling sites**. Arrows indicate the direction of the currents (from Liu et al., 2007). ZMCC, Zhe-Min coastal current; TWWC, Taiwan Warm Current; YSWC, Yellow Sea Warm Current; YSCC, Yellow Sea Coastal Current; CRDW, Changjiang River diluted water.

Collectively, the eastern China marginal seas (ECMS), including the Bohai Sea, the Yellow Sea, and the East China Sea (ECS), compose one of the largest marginal seas in the world (Figure 1; Song, 2011). The most characteristic feature of ECMS is the existence of isolated mud patches on sandy sediments. ECMS receive ~480 and 1000 Mt/a (million ton/annual) sediment from the Yellow River and the Changjiang River, respectively, representing ~10% of global sediment discharge (Xu et al., 2012). These sediments together with small amount of sediments from other adjacent rivers and open sea form several mud zones controlled by estuarine processes, tidal currents, and shelf circulation (Liu et al., 2007; Hu et al., 2013). The Changjiang Estuary mud zone and the Zhe-Min mud zone are two inner-shelf muds, and their sedimentation rates are higher vs. those of the distal Cheju Island mud zone and South Yellow Sea (SYS) mud zone. The geographic location, sedimentary sources, and influence of ocean currents of the mud zones in ECMS are different from each other, different microbial community may therefore exist in different locales.

As prominent examples of the river-dominated ocean margins, ECMS have received considerable attention concerning AOA and AOB populations (e.g., Dang et al., 2008; Zheng et al., 2013, 2014; Chen et al., 2014b); however, the community composition and differentiation of ammonia-oxidizing prokaryotes in different mud zones of the ECMS are still unknown. Furthermore, most of these studies have focused on the community compositions of ammonia-oxidizing prokaryotes in surface sediments along environmental gradients. A field survey of AOB assemblages has revealed a vertical change of abundance of AOB in the ECS sediment (Chen et al., 2014b). It therefore could be inferred that the community compositions of other ammonia-oxidizing prokaryotes might also change vertically. The vertical profile of AOA and AOB would enable a better understanding of their ecological services in the marine benthic environment.

Ammonia monooxygenase (AMO), the key enzyme in ammonia oxidation, is common in AOA and AOB. The *amoA* gene encoding the α-subunit of AMO provides greater resolution for discerning closely-related but distinct ammonia-oxidizing strains when compared with the 16S rRNA gene (Rotthauwe et al., 1997), and has been commonly used as a phylogenetic marker to study the populations of AOA and AOB (Junier et al., 2010). In this study, the horizontal and vertical community compositions and abundance of ammonia-oxidizing prokaryotes in sediments of mud areas of the ECMS and their niche differentiation were investigated by using archaeal and bacterial *amoA* genes clone libraries and quantitative PCR (qPCR). In addition, key environmental factors that likely shape the community composition and abundance of ammonia-oxidizing prokaryotes were identified based on statistical analysis. We investigated different mud zones at different depths to see if they might possess distinct AOA and AOB populations, and if so, how environmental factors contribute to the observed community shifts.

## Materials and methods

### Site description and sampling

Sampling was conducted on board the R/V *Dongfanghong 2* in July, 2013. Sediment cores from five sites, with the water depth ranging from 24 to 83m, in four different mud zones of ECMS (Figure 1) were collected using a stainless steel box-sampler. ECS01 is located in the Changjiang Estuary mud zone, where the principal origin of sediment is the Changjiang River, deposited under the effect of its diluted water, tidal actions, and coastal currents (Liu et al., 2007; Hu et al., 2012); sedimentation rates in this mud zone range from ~2.0 to 6.0 cm/y (centimeters/year) (Chen et al., 2004). ECS02 is located in the Zhe-Min mud zone, which is a mud wedge along the ECS inner shelf with sedimentation rates ranging from 0.4 to 1.2 cm/y (Huh and Su, 1999). Its main sedimentary sources are suspended sediments from the Changjiang River and resuspended sediments from the Changjiang Estuary mud zone; sediments are transported by the southward Zhe-Min coastal current in winter when the strength of the northward Taiwan Warm Current declines (Liu et al., 2007). ECS03 is from the distal Cheju Island mud zone, a mud patch located to the southwest of Cheju Island with sedimentation rates ranged from 0.1 to 0.5 cm/y (Yang et al., 2003; Lim et al., 2007). Sediments of this mud zone are derived from both the Changjiang River and the Old Yellow River delta (Liu et al., 2003). SYS01 and SYS02 are located in the SYS mud zone, which is formed by a counter-clockwise gyre with the northwestward Yellow Sea Warm Current in winter and southward Yellow Sea Coastal Current along the China's eastern coasts. Sedimentation rate in the SYS mud zone is < 0.1 cm/y. The muddy sediments in this area are mainly supplied by the modern and/or old Yellow River (Yang and Youn, 2007; Hu et al., 2013).

At each site, two PVC tubes (7.5-cm internal diameter) were carefully inserted into the box-sampler to take sub-cores without disturbing the surface sediments. One sub-core was immediately sectioned at 1-cm intervals using a stainless-steel cutter, and these samples were stored at −20°C on board the research vessel (about 20 days) and subsequently transferred to -80°C in laboratory prior to organic carbon (OC) analysis and DNA extraction. Pore waters were collected at cm-scale resolution throughout the full length of the other sub-core using Rhizon samplers inserted through pre-drilled holes in the core tube and attached to vacuum test tubes. After collection, pore water was poisoned by HgCl_2_ and stored at 4°C for subsequent analysis of dissolved inorganic nutrients.

### Determination of environmental parameters

Total OC (TOC), total nitrogen (TN), stable carbon, and nitrogen isotopes (δ^13^C and δ^15^N) were analyzed following the method of Yao et al. (2014). Dissolved inorganic nutrients (NO_3−_, NO${}_{2}^{-}$, NH${}_{4}^{+}$, PO${}_{4}^{3-}$, and SiO${}_{3}^{2-}$) in sediment pore waters were determined colorimetrically on a nutrient auto-analyzer (AA3, Seal Analytical Ltd., UK). Salinity of the bottom water at each site was obtained by CTD (SBE 25 SEALOGGER, Sea-Bird Electronic Inc., USA). Detection limits for NO${}_{3}^{-}$, NO${}_{2}^{-}$, NH${}_{4}^{+}$, PO${}_{4}^{3-}$, SiO${}_{3}^{2-}$, and DO were 0.02, 0.01, 0.04, 0.02, 0.01, and 1–2 μM L^−1^, respectively. Analytical uncertainty for all dissolved nutrients in replicate samples was < 5–10%.

### DNA extraction, *amoA* amplification, cloning, and sequencing

DNA was extracted using Power Soil DNA Isolation Kit (Mo Bio, USA) according to the manufacturer's suggested protocol. DNA concentration was measured using a ND-2000 Nanodrop spectrophotometer (Thermo Fisher Scientific, USA), and the remaining DNA was stored at −80°C. Amplification and *amoA* clone library construction followed previously described procedures (Zheng et al., 2014). DNA extracted from three sediment layers (0–1, 12–13, and 32–33 cm, referred to as -S, -M, and -B, respectively) was used as template DNA. The primer sets and programs for amplification of *amoA* fragments are shown in Table 1; pUCm-T vectors were used for cloning.

**Table 1:** **Primers and PCR conditions used for the PCR amplification**.

**Target gene**	**Primer (5′-3′)**		**Length of amplicon (bp)**	**Thermal profile**	**References**
AOA *amoA*	Arch-*amoA*F	STAATGGTCTGGCTTAGACG	635	5 min at 95°C, followed by 30 cycles of 45 s at 95°C, 60 s at 53°C and 60 s at 72°C, then, 10 min at 72°C. (PCR)	Francis et al., 2005
	Arch-*amoA*R	GCGGCCATCCATCTGTATGT		95°C for 30 s, followed by 40 cycles of 30 s at 95°C, 60 s at 56°C, and 60 s at 72°C. (qPCR)	
AOB *amoA*	*amoA*-1F	GGGGTTTCTACTGGTGGT	491	5 min at 95°C95°C, followed by 30 cycles of 45 s at 95°C95°C, 90 s at 56°C56°C and 60 s at 72°C72°C, then, 10 min at 72°C72°C. (PCR)	Rotthauwe et al., 1997
	*amoA*-2R	CCCCTCKGSAAAGCCTTCTTC		95°C for 30 s, followed by 40 cycles of 30 s at 95°C, 60 s at 58°C, and 35 s at 72°C. (qPCR)	

### Real-time qPCR

To quantify AOA and AOB, DNA extracted from sediment samples at eight depths (0–1, 1–2, 2–3, 3–5, 7–8, 12–13, 22–23, and 32–33 cm, referred to as –0, –1, –2, –3, –5, –10, –20, and –30, respectively) was used as template for qPCR. Preparation of qPCR standards and performance of qPCR analysis was conducted according to the procedure of Zheng et al. (2014) with minor modification. Briefly, the 20 μl qPCR mixture contained 10 μl of SYBR Premix Ex Taq II (2×), 0.4 μl of ROX Reference Dye II (50×), 0.8 μl of each primer (10μM), and 2μl template DNA. The qPCR thermal cycling steps were shown in Table 1. In all experiments, negative controls lacking template DNA were subjected to the same qPCR procedure to detect and exclude any possible contamination.

The consistency of the qPCR assay was confirmed by the strong linear inverse relationship between the threshold cycle and the log value of gene copy number for both primer sets (*r*^2^ = 0.9988 for AOB and 0.9987 for AOA). The amplification efficiencies were 93.82 and 92.88% for AOB and AOA, respectively. In addition, melting curve analyses showed only one observable peak at melting temperature (85.4 for AOB and 84.5 for AOA), and no detectable peaks associated with primer-dimer artifacts or other nonspecific PCR amplification products were observed after gel electrophoresis.

### Phylogenetic and statistical analyses

The *amoA* gene sequences were edited using the DNAstar software package (http://www.dnastar.com). The existence of potential chimeras was checked using the CHECK CHIMER program of the Ribosomal Database Project (Cole et al., 2007). All of the sequences were aligned using the Clustal X program (Thompson et al., 1997) and grouped into different operational taxonomic units (OTUs) with a 5% distance cut-off using the DOTUR program (Schloss and Handelsman, 2005). Diversity indices, including Shannon–Wiener (*H*), Simpson (*D*), species richness estimator Chao1, species evenness (*J*), and abundance-based coverage estimator (ACE) were also performed using DOTUR. In addition, data with respect to Shannon–Wiener index and *amoA* abundance of AOA and AOB in a serial studies (Dang et al., 2008, 2009, 2010a,b, 2013; Park et al., 2008; Cao et al., 2011, 2012; Zheng et al., 2013, 2014; Chen et al., 2014b) were retrieved and compared in order to get a comprehensive insight into the diversity and abundance of AOA and AOB along surface sediments of the west Pacific marginal seas. Reference sequences were selected using the BLASTn tool (http://blast.ncbi.nlm.nih.gov/Blast.cgi). Neighbor-joining (NJ) phylogenetic trees were generated by the MEGA software (Tamura et al., 2011) with the K2 + G model using 1000 bootstrap replicates. The tree was built with a final alignment of 587 columns for AOA and 449 columns for AOB, respectively.

Canonical correspondence analysis (CCA) was executed in CANOCO 5 for Windows (Microcomputer Power, Ithaca, New York, United States) to correlate the composition of AOA and AOB communities with environmental factors. The distribution and abundance matrix of OTUs for both AOA and AOB were square root transformed and then used as species data for the CCA analysis. Because data for dissolved inorganic nutrients in ECS03 were lacking, samples from only four sites were included in the CCA analysis. Community classifications of AOA and AOB were performed using principal coordinate analysis (PCoA) conducted via online software (Fast UniFrac, http://unifrac.colorado.edu). One-way ANOSIM (Analysis of similarities) was conducted in PRIMER 6 (Plymouth Routines In Multivariate Ecological Research) using the Bray Curtis resemblance matrix calculated from square root transformed OTU tables to determine the significant differences between samples in different mud zones.

### Nucleotide sequence accession number

All the archaeal and bacterial *amoA* sequences reported in this study have been deposited in the GenBank database under accession numbers KM595340–KM596501.

## Results

### Environmental parameters

The TOC (%) content of each site ranged from 0.55 ± 0.06 at station ECS03 to 0.90 ± 0.07 at SYS01 (Table S1). The C/N ratios in sediments at SYS stations were slightly higher than those of ECS stations (*P* < 0.01, Wilcoxon rank sum test). Compared to other stations, the δ^13^C showed more depleted value at station ECS01 (*P* < 0.001). For δ^15^N_TN_, the lowest value was 2.84 ± 0.59 at ECS01, while the highest was 5.50 ± 0.25 at ECS03 (Table S1). SiO${}_{3}^{2-}$ ranged from 235.8 ± 28.5 to 399.0 ± 36.8 and its concentration varied within a larger range at station ECS01 than at other stations (Figure S1B5). The concentration of NH${}_{4}^{+}$ was highest (*P* < 0.05) and increased with depth from 153.0 to 617.5 μmol L^−1^ at station ECS01 (Table S1; Figure S1B3). In contrast to SiO${}_{3}^{2-}$ and NH${}_{4}^{+}$, the lowest concentrations of PO${}_{4}^{3-}$ were recorded at station ECS01 (*P* < 0.05). In addition, the concentration of NO${}_{3}^{-}$ was significantly higher in SYS than in ECS stations (*P* < 0.01). Details about the distribution of organic parameters of sediments and inorganic nutrients in sediment pore waters are shown in Figure S1. The salinity of bottom water at SYS01, SYS02, ECS01, ECS02, and ECS03 was 30.3, 31.1, 33.4, 34.4, and 32.3%0, respectively.

### Diversity of archaeal and bacterial *amoA* assemblages

The number of sequenced clones varied among samples, as it was determined according to the calculated diversity estimators. If there was no diversity change after more sequences were added to the dataset, we did not continue to increase the seqeuncing depth. A total of 613 archaeal *amoA* sequences were obtained, and were classified into 83 OTUs based on a cut-off threshold of 5% distance in similarity. Each individual archaeal *amoA* clone library possessed 3–24 OTUs; the distribution of relative abundance of OTUs is shown in Figure S2A. Interestingly, a unique OTU type existed in almost each sample, and none of the OTUs were shared by all the samples. The Shannon-Wiener and Simpson indices showed that the diversity of archaeal *amoA* was highest in ECS01-B and lowest in SYS01-B. The diversity of archaeal *amoA* in all samples of ECS was significantly higher than that observed at sites of SYS (*P* < 0.05, Table 2).

**Table 2:** **Biodiversity and predicted richness of the archeal *amoA* sequences and the bacterial *amoA* sequences**.

**Sample**	**AOA**								**AOB**							
	**No. of clones**	**No. of OTU^a^**	***C* (%)**	***H***	**1*D***	***J***	***S_*ACE*_***	***S*_*Chao*1_**	**No. of clones**	**No. of OTU^a^**	***C* (%)**	***H***	**1*D***	***J***	***S_*ACE*_***	***S*_*Chao*1_**
SYS01-S	77	15	94.81	2.12	5.92	0.78	17.80	16.20	72	14	95.83	2.17	6.83	0.82	16.47	14.60
SYS01-M	24	7	91.67	1.57	4.06	0.81	8.52	7.25	19	6	84.21	1.23	2.51	0.69	9.75	7
SYS01-B	18	3	93.75	0.43	1.27	0.39	0	4	21	6	85.71	1.48	4.38	0.83	9.1	9
SYS02-S	77	19	87.01	2.04	4.58	0.69	35.74	25.43	66	13	95.45	2.24	8.61	0.87	15.03	14.5
SYS02-M	24	6	83.33	1.19	2.60	0.66	21.19	12	19	8	84.21	1.92	8.55	0.92	9.90	9
SYS02-B	18	4	88.89	0.76	1.66	0.55	7	4.5	18	4	100	1.30	4.02	0.94	4	4
ECS01-S	75	19	89.33	2.34	7.50	0.79	26.07	23.67	69	12	92.75	1.93	5.59	0.78	19.27	17
ECS01-M	35	14	80.00	2.30	8.881	0.87	25.99	19.25	19	3	94.74	0.81	2.1	0.74	4.11	3
ECS01-B	41	19	82.93	2.72	15.47	0.92	26.97	21.33	20	4	95	1.03	2.47	0.74	4.83	4
ECS02-S	78	24	84.62	2.57	9.1	0.818	50.08	32.25	81	7	97.53	0.93	1.73	0.4	8.95	7.33
ECS02-M	15	6	86.67	1.62	5.83	0.90	7.35	6.33	19	2	94.74	0.21	1.12	0.30	0	2
ECS02-B	23	9	86.96	2.07	10.12	0.94	10.43	10.5	20	5	85	1.05	2.26	0.65	13.25	8
ECS03-S	73	18	93.15	2.40	7.87	0.83	22.74	19.43	69	8	95.65	1.17	2.10	0.56	10.26	11
ECS03-M	20	4	100	1.28	3.80	0.92	4	4	18	1	100	0	1	1.00	0	1
ECS03-B	16	3	93.75	0.86	2.1	0.78	3.58	2	19	2	94.74	0.21	1.12	0.30	0	2
Total	613	83		3.38	16.98	0.76	130.57	116	549	37		2.43	6.26	0.67	45.53	43.43

a *OTUs of amoA sequences at the cutoff of 5% were determined using the Dotur program. C, coverage of the constructed clone libraries; H, Shannon-Weiner index; D, Simpson index; J, evenness index; S_ACE_ and S_Chao1_, richness estimators. -S, -M, and -B referred to depths at surface (0–2 cm), middle (12–13 cm), and bottom (32–33 cm) layers, respectively*.

Based on the 549 bacterial *amoA* sequences obtained from 15 clone libraries, 36 OTUs occurred with a cut-off threshold of 5% distance in similarity. The diversity of bacterial *amoA* in SYS02-S was the highest according to diversity indices. The diversity of bacterial *amoA* in the surface sediment was higher than those of the other two layers among all the sites except for ECS02 (Table 2).

**Figure 2:**
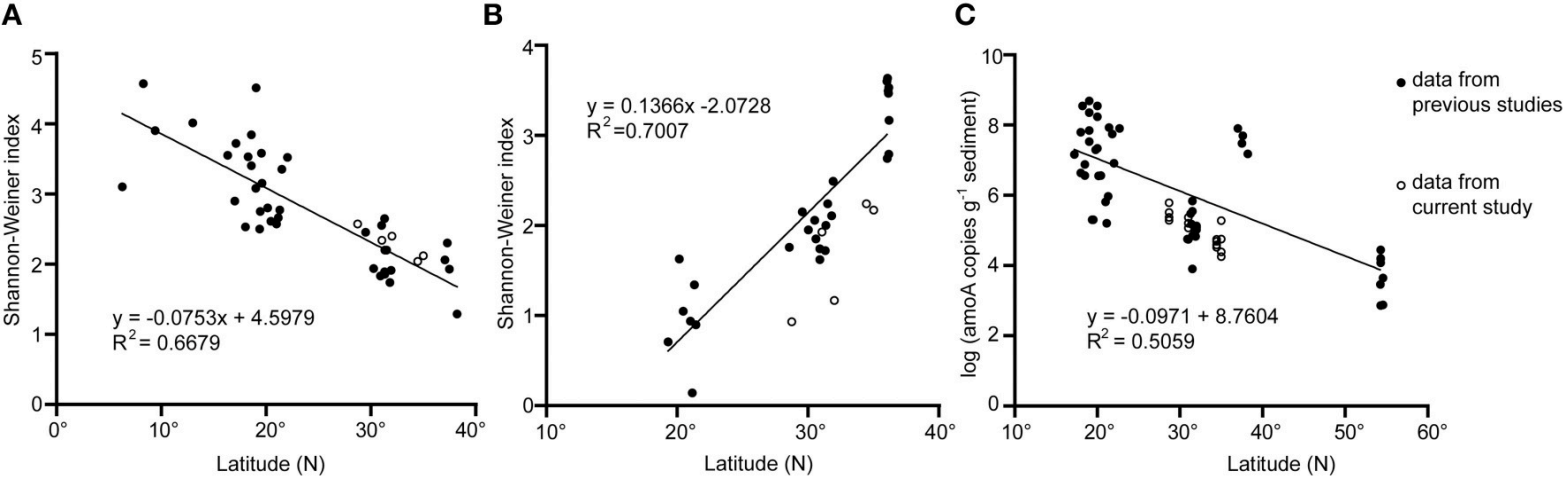
**Diversity of archaeal and bacterial *amoA* and abundance of archaeal *amoA* changed along latitude in sediments of the western Pacific marginal seas**. **(A)** diversity of AOA *amoA* gene; **(B)** diversity of AOB *amoA* gene; **(C)** abundance of AOA *amoA* gene. Data were obtained from the present study and Dang et al. (2008, 2009, 2010a,b, 2013), Park et al. (2008); Cao et al. (2011, 2012); Zheng et al. (2013, 2014), and Chen et al. (2014b).

The high coverage values in each sample (more than 80%) verified the reliability of the clone libraries, although a higher sequencing depth might increase the OTU numbers of some samples according to the rarefaction curves (Table 2, Figure S3). The diversity of the entire set of bacterial *amoA* was lower than that of archaeal *amoA* based on the values of the Shannon-Winner index (*P* < 0.05; Table 2). For samples in SYS, the diversity level of bacterial *amoA* was comparable to, or higher than, that of archaeal *amoA* in the same sample, while for samples in ECS, the diversity of bacterial *amoA* is lower than that of archaeal *amoA* (*P* < 0.001). Broadly, the diversity of AOA from the surface sediment of SYS was generally higher than that of the East Korea Sea but lower than that of ECS (Park et al., 2008), and all of them were lower than those of SCS and the tropical West Pacific Continental Margin (Dang et al., 2009, 2013; Cao et al., 2011; Figure 2A). The diversity of AOB in surface sediments of SYS was higher than those of the other three mud zones in ECS and SCS (Cao et al., 2011, 2012), and was lower than that of the Jiaozhou bay (Dang et al., 2010a) (Figure 2B).

### Phylogenetic distribution of AOA and AOB

**Figure 3:**
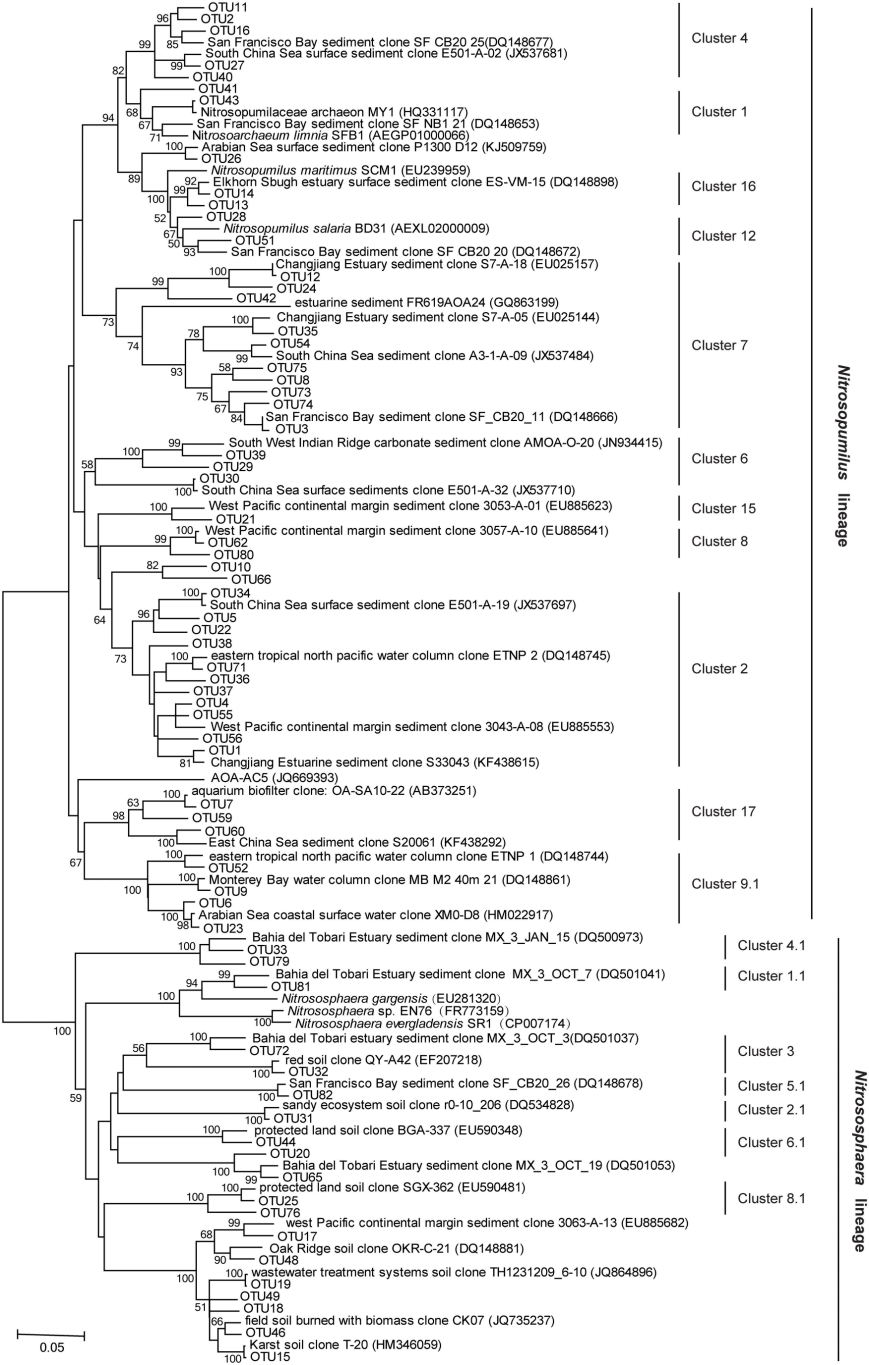
**Distance-based neighbor-joining phylogenetic tree of the archaeal *amoA* sequences recovered from mud deposits of the eastern China marginal seas and their closest matches in GenBank**. Bootstrap support values >50% (1000 replicates) are shown.

**Figure 4:**
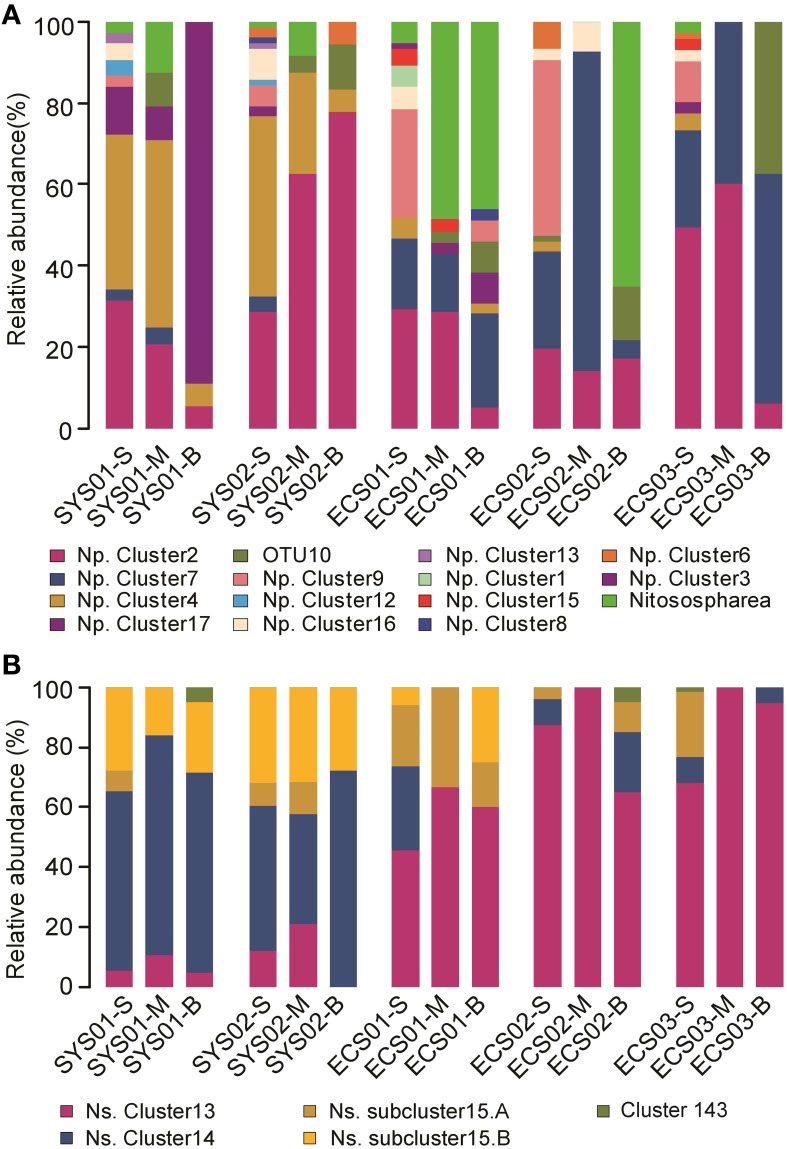
**Distribution and relative abundance of phylogenetic AOA and AOB groups in mud deposits of the China Eastern Marginal Seas**. **(A)**, AOA; **(B)**, AOB. Np., *Nitrosopumilus*; Ns., *Nitrosospira*.

All the archaeal *amoA* sequences obtained in this study were phylogenetically grouped into two lineages: a *Nitrosopumilus* lineage (previously also referred to as marine sediment/water lineage; Cao et al., 2011) and a *Nitrososphaera* lineage (previously also referred to as soil/sediment lineage; Cao et al., 2011; Figure 3). Of the total archaeal *amoA* sequences, 89.6% were affiliated to the *Nitrosopumilus* lineage. The closest matching GenBank sequences for this lineage were originally retrieved from estuarine, coastal, and marine environments, including sediments of San Francisco Bay, Elkhorn Slough estuary, Changjiang Estuary, East China Sea, South China Sea, West Pacific continental margin, Monterey Bay, Gulf of Mexico, and Arabian Sea water column. According to the clustering of Pester et al. (2012), the *Nitrosopumilus* lineage in this study was further classified into 10 known clusters (Cluster 1, 2, 4, 6, 7, 8, 9.1, 12, 15, and 16) and one novel cluster which was tentatively named as Cluster 17. Their relative abundance in each sample is shown in Figure 4A. Sequences of Cluster 17 were mainly obtained from SYS01 (27/35) and were 88.1–93.8% identical among each other. All of the sequences in *Nitrosopumilus* Clusters 9, 12, and 16 occurred in surface sediments, with the exception of two found in ECS01-B and one found in ECS02-M. OTUs in *Nitrosopumilus* Cluster 7 were mainly found in samples from ECS (*P* < 0.001). The predominant OTU of this cluster was OTU3 with almost all sequences but one from the mud deposits in ECS. In contrast, OTUs in *Nitrosopumilus* Cluster 4 (such as OTU2) were mainly obtained from the surface and middle layers in SYS (*P* < 0.001). Sequences in *Nitrosopumilus* Cluster 2 were widespread in all samples, but sequences of OTU1 in this cluster were mainly discovered in SYS (*P* < 0.05) while those of OTU4 and OTU5 were mainly enriched in ECS01-S and ECS03-S, respectively. OTU10 affiliated to the *Nitrosopumilus* lineage was discovered from the mid and/or bottom sediments of all the five sites but absent from any surface sediments. It showed a low identity value (93%) to the top hit sequence in GenBank and might represent a new clade as evidenced by the phylogenetic topology (Figure 3). Sequences in the *Nitrososphaera* lineage were mainly related to terrestrial and estuarine environments, and most of them were discovered in ECS01-M, ECS01-B, and ECS02-B.

The bacterial *amoA* sequences were phylogenetically grouped into the *Nitrosospira* lineage and the *Nitrosomonas* lineage (Figure S4). The *Nitrosospira* lineage, which contained 99.5% of the total bacterial *amoA* sequences, was the dominant group of AOB across all the studied sites. OTUs in this lineage were not affiliated within any *amoA* sequence cluster defined by cultured AOB (Purkhold et al., 2003), but affiliated within three novel clusters (Cluster 13, 14, and 15) defined by Dang et al. (2010a) with sequences from diverse estuarine, coastal, and deep-sea environments. The phylotypes of bacterial *amoA* in SYS and ECS were different. Cluster 13 is the dominant group in samples of ECS01, ECS02, and ECS03 (44.9–100%, *P* < 0.05), but the relative abundance of it in the two sites of SYS is lower (0–21.1%; Figure 4B). Sequences in Cluster 14 were mainly recovered from SYS01 and SYS02 (76.6%, *P* < 0.01). Cluster 15 could be divided into two subclusters, A and B. OTUs belonging to Cluster 15 were predominant in the surface sediment across all sites (*P* < 0.01), but the abundance of subclusters varied in different stations. Subcluster B mainly appeared in SYS01 and SYS02 (*P* < 0.01) while subcluster A was mainly in ECS01 (Figure 4B). The phylotypes of bacterial *amoA* in SYS01 and SYS02 were similar. OTU1 in Cluster 13, OTU2 and OTU4 in Cluster 14, and OTU5 in Cluster 15 were shared among the six samples in the two sites. Although these four OTUs represented only 11.1% of all the OTUs identified, they accounted for 63.16–84.21% of the total sequences in each sample (Figure S2B). Cluster 143 only appeared in SYS01-B, ECS02-B, and ECS03-S, and only included three sequences.

### Community classification and spatial distribution of *amoA* sequences

The results of the fast UniFrac all-environment significance test (*P* < 0.01) and the *P*-test (*P* < 0.001) indicated that both AOA and AOB communities varied significantly in different samples.

The weighted UniFrac PCoA analysis grouped AOA assemblages of the 15 samples into three clusters (Cluster I: ECS01-B, ECS01-M, and ECS02-B; Cluster II: ECS01-S, ECS02-S, ECS02-M, ECS03-S, ECS03-M, and ECS03-B; Cluster III: samples from SYS01 and SYS02; Figure S5A). Samples from SYS01 and SYS02 were significantly separated from ECS01, ECS02, and ECS03 along the first principal coordinate (ANOSIM, *R* = 0.497, *P* < 0.005); meanwhile, Cluster I could be separated from Cluster II along the second principal coordinate (P2) (Figure S5A).

Consistent with AOA, the AOB assemblages from SYS02 and SYS01 were grouped in a cluster which are significantly separated from ECS01, ECS02, and ECS03 along the first principal coordinate (P1) of the UniFrac PCoA analysis (ANOSIM, *R* = 0.942, *P* < 0.005). The AOB assemblages from samples of ECS02 and ECS03 were more similar to each other, and were separated from those of ECS01 (Figure S5B).

### Abundance of archaeal and bacterial *amoA*

**Figure 5:**
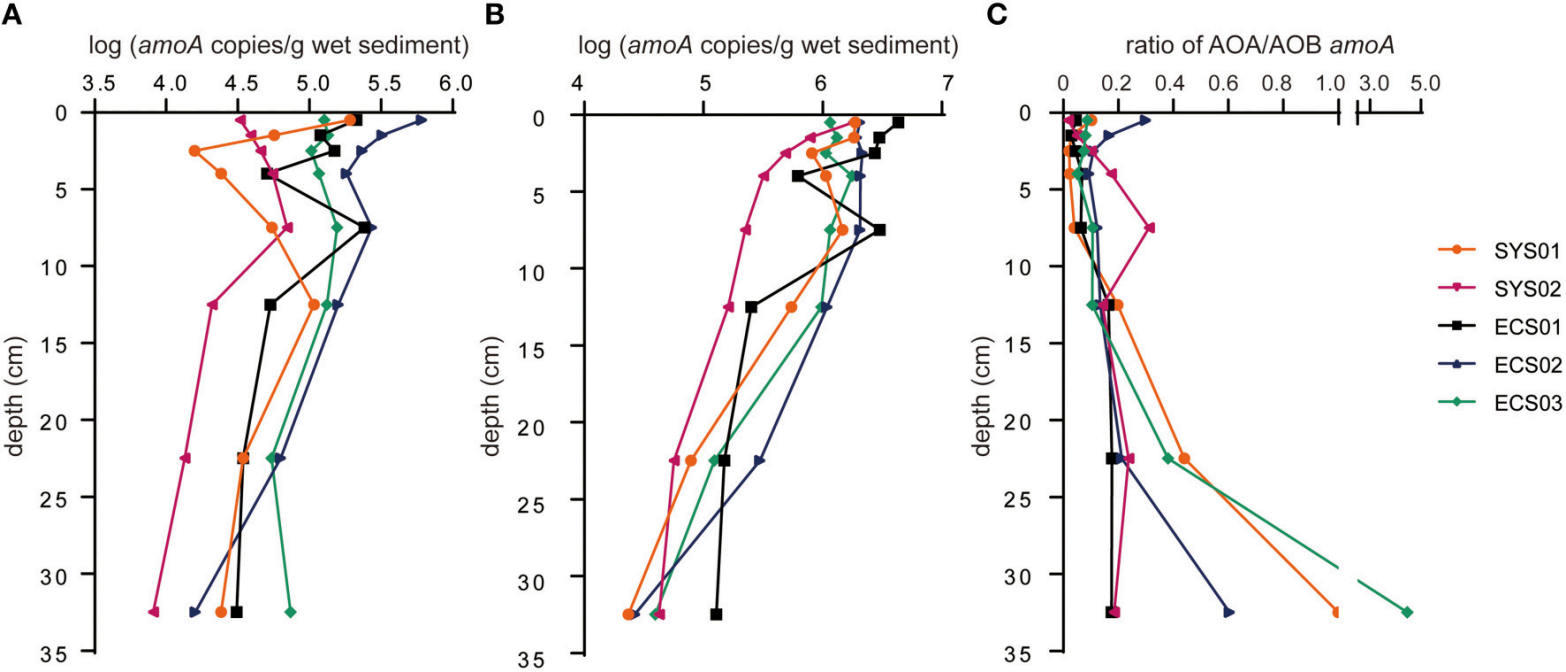
**Quantitative analysis of *amoA* for AOA and AOB in mud deposits of the eastern China marginal seas**. **(A)**, AOA; **(B)**, AOB; **(C)**, ratio of archaeal to bacterial *amoA*. Error bars represent standard deviations of triplicate analyses. -0, -1, -2, -3, -5, -10, -20, and -30 represent samples at the depth of 0–1, 1–2, 2–3, 3–5, 7–8, 12–13, 22–23, and 32–33 cm, respectively.

The abundance of *amoA* sequences ranged from 8.11 × 10^3^ to 6.09 × 10^5^ copies/g sediment (wet weight) for AOA and from 2.34 × 10^4^ to 4.31 × 10^6^ copies/g sediment (wet weight) for AOB in different samples (Figures 5A,B). The highest copy number of archaeal *amoA* was found in ECS02-0, while that of bacterial *amoA* was in ECS01-0. Most layers of ECS02 showed higher archaeal *amoA* abundance than the same layer of other sites, and the same situation was found in the bacterial *amoA* abundance of ECS01.

The abundance of both archaeal and bacterial *amoA* in the samples of the first five centimeters was fluctuant except for SYS02, where the abundance of bacterial *amoA* reduced with the increase of the depth while archaeal *amoA* showed a reverse trend. Generally, the ratio of archaeal *amoA* to bacterial *amoA* increased with the increase of depth (Figure 5C). The abundance of bacterial *amoA* was greater than that of archaeal *amoA* in the same sample (except for SYS01-30 and ECS03-30). Together with the results of previous studies, the abundance of archaeal *amoA* in surface sediments of the western pacific marginal seas decreased with the increase of latitude (Figure 2C), whereas the abundance of bacterial *amoA* did not have this correlation (data not shown).

### Correlation of ammonia oxidizers with environmental variables

**Figure 6:**
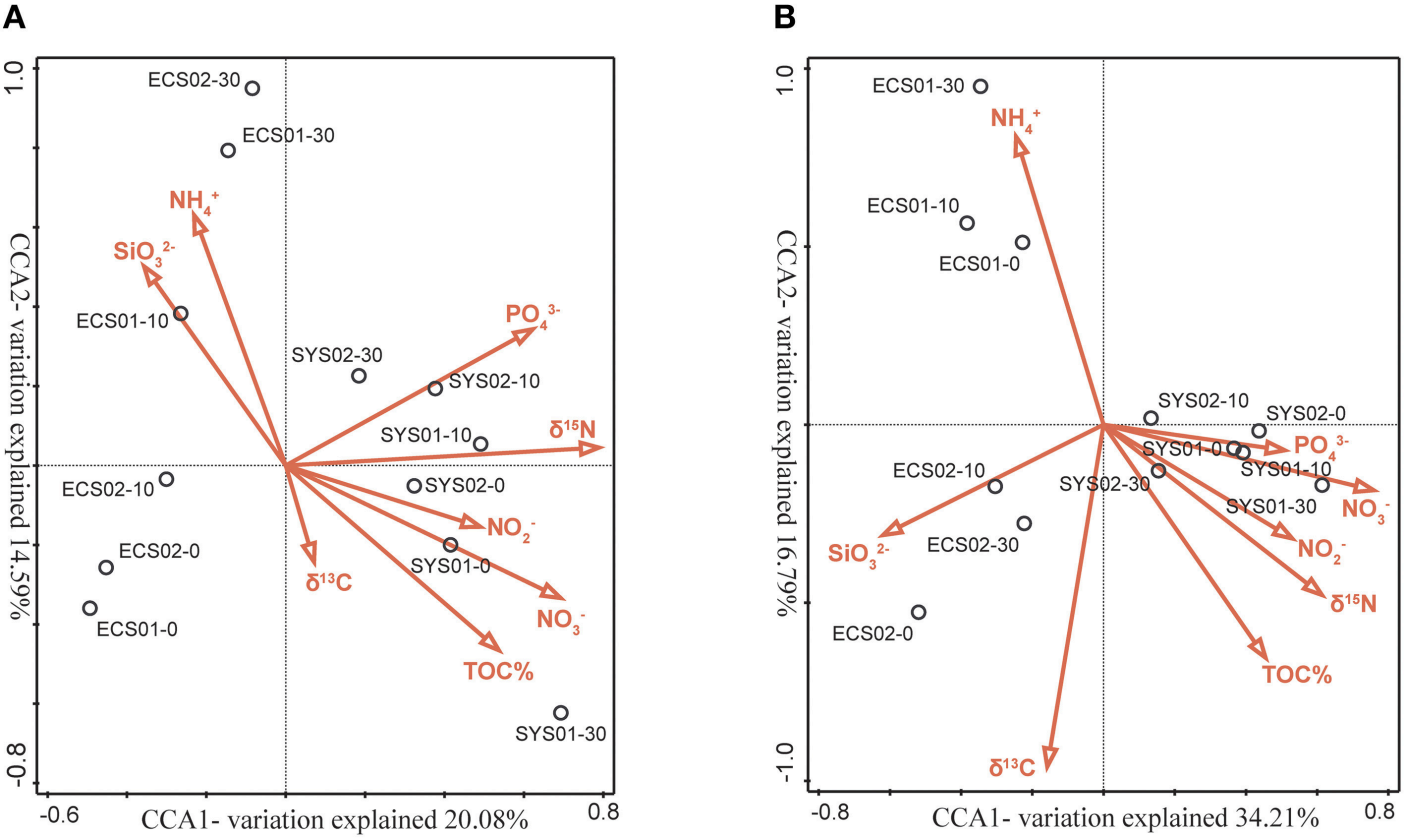
**Ordination plots generated by CCA based on the weighted OTU data**. The relationship between the AOA **(A)** and AOB **(B)** *amoA* community compositions in mud deposits of the eastern China marginal seas and environmental parameters were analyzed. Correlations between environmental variables and CCA axes are represented by the lengths and angles of arrows (environmental-factor vectors).

CCA analysis was used to identify the key environmental factors that contributed to the heterogeneous distribution of the sediment AOA and AOB assemblages (Figures 6A,B). Both AOA and AOB communities of samples in SYS were grouped together and their distribution was positively correlated with PO${}_{4}^{3-}$, NO${}_{3}^{-}$, NO${}_{2}^{-}$ and δ^15^N_TN_ (Figures 6A,B).

The first two CCA axes explained 34.67% of the total unconstrained variance in the AOA community composition and 44.40% of the cumulative variance in the genotype–environment relationship. The AOA assemblages of samples in ECS01 and ECS02-B were grouped together and their distribution was positively correlated with NH4^+^ and negatively correlated with TN and TOC. δ^15^N_TN_ (*P* = 0.002, *F* = 2.0; 499 Monte Carlo permutations), SiO${}_{3}^{2-}$ (*P* = 0.008, *F* = 1.8; 499 Monte Carlo permutations) and NH${}_{4}^{+}$ (*P* = 0.044, *F* = 1.7; 499 Monte Carlo permutations) contributed at least marginally significantly to the genotype–environment relationship, and explained 21.0, 17.5, and 15.9% of the total variance, respectively.

The first two CCA axes explained 51.0% of the total unconstrained variance in the AOB community composition and 60.07% of the cumulative variance in the genotype–environment relationship. Unlike AOA, the AOB assemblages of samples from the same site of ECS grouped together. The distribution of samples in ECS01 was positively correlated with NH${}_{4}^{+}$ and negatively correlated with C/N and δ^15^N_TN_ while that of samples in ECS02 was positively correlated with SiO${}_{3}^{2-}$. NO${}_{3}^{-}$ (*P* = 0.004, *F* = 3; 499 Monte Carlo permutations) and δ^13^C (*P* = 0.006, *F* = 2.5; 499 Monte Carlo permutations) contributed significantly to the genotype–environment relationship, and explained 27.1 and 19.5% of the total variance, respectively.

Correlation of *amoA* abundance and diversity with environmental variables was also detected (Table 3). The archaeal *amoA* copy numbers were positively correlated with SiO${}_{3}^{2-}$ (*r* = 0.3584, *P* = 0.0440, *n* = 32) and negatively correlated with PO${}_{4}^{3-}$ (*r* = −0.5121, *P* = 0.0027, *n* = 32) while the bacterial *amoA* copy numbers were positively correlated with NO${}_{3}^{-}$ (*r* = 0.4777, *P* = 0.0057, *n* = 32) and negatively correlated with SiO${}_{3}^{2-}$ (*r* = −0.4540, *P* = 0.0091, *n* = 30). The ratio of archaeal to bacterial *amoA* copy numbers was significantly correlated with SiO${}_{3}^{2-}$ (*r* = 0.5923, *P* = 0.0004, *n* = 32).

**Table 3:** **Statistical analysis of physicochemical parameters and the diversity and abundance of *amoA* for AOA and AOB in this study**.

**Parameter**	**Pearson moment correlation^a^**										
	**OTU**		**Shannon index**		**Simpson index**		**Chao**		**Abundance**		
	**AOA**	**AOB**	**AOA**	**AOB**	**AOA**	**AOB**	**AOA**	**AOB**	**AOA**	**AOB**	**AOA/AOB**
TOC%	0.1293	0.6192^*^	0.0288	0.6643^*^	−0.1626	0.6247^*^	0.2162	0.5479^*^	−0.0141	0.1264	−0.3241
TN%	0.3170	0.6085^*^	0.2526	0.5818^*^	0.0367	0.4869	0.3610	0.5340^*^	0.1083	0.3006	−0.2630
C/N	−0.3194	0.2357	−0.4404	0.3896	−0.4770	0.4957	−0.2021	0.2244	−0.2057	−0.2477	−0.0839
δ13C%0	−0.3683	−0.2776	−0.4262	−0.3966	−0.5009	−0.3200	−0.3447	−0.2591	0.2643	−0.1870	0.2082
δ15N%0	−0.4484	−0.1562	−0.4637	−0.2172	−0.4124	−0.1664	−0.5371^*^	−0.2074	−0.1707	−0.1468	0.1582
NO${}_{3}^{-}$	0.1039	0.7533^*^	−0.0743	0.6609^*^	−0.3548	0.6517^*^	0.0736	0.6099^*^	−0.0356	0.4777^*^	0.0761
NO${}_{2}^{-}$	0.2953	0.5923^*^	0.2328	0.4184	−0.0286	0.4018	0.2535	0.4660	−0.0031	0.3054	0.0620
NH${}_{4}^{+}$	0.2544	−0.3564	0.4007	−0.2303	0.6565^*^	−0.2445	0.2051	−0.3755	−0.2254	−0.1057	−0.2251
PO${}_{4}^{3-}$	−0.7808^*^	−0.4661	−0.6496^*^	−0.2973	−0.5096	−0.0133	−0.7436^*^	−0.5119	−0.5121^*^	−0.0958	−0.0554
SiO${}_{3}^{2-}$	0.1160	−0.6582^*^	0.3539	−0.7668^*^	0.5629	−0.6246^*^	0.0937	−0.7535^*^	0.3584^*^	−0.4540^*^	0.5923^*^

a *Pearson moment correlation (r). Asterisks indicate P < 0.05*.

## Discussion

### Predominant lineages and community compositions of AOA and AOB

The majority of bacterial and archaeal *amoA* sequences obtained in this study fell within *Nitrosospira* (99.5%) and *Nitrosopumilus* lineages (89.6%), respectively, with their reference sequences isolated from marine and estuarine environments. Sequences in the *Nitrosopumilus* lineage were mainly affiliated to Clusters 2, 4, and 9 (known as the “marine cluster”), or to the Cluster 7 (known as the “estuarine cluster”), according to the phylogenetic classification proposed by Pester et al. (2012). A negligible number of sequences in the *Nitrosomonas* lineage of AOB and the *Nitrososphaera* lineage of AOA were discovered; their reference sequences originated from soil, upper estuarine, and coastal areas with low salinity and impacts from terrestrial disturbances (Francis et al., 2003; Bernhard et al., 2005; Mosier and Francis, 2008). These results indicated that the ocean-dominant lineages may be better adapted to mud sediments of the marginal sea where the salinities of bottom water were at the range of ocean salinities (30.3–34.4%).

### Niche differentiation of ammonia-oxidizing prokaryotes communities

Ammonia-oxidizing prokaryotes communities were found to vary with sediments at different depths. It was interesting to note that OTU10 of AOA mainly occurred in the mid and bottom sediment layers, where DO and bottom disturbance were lower. Although it has been suggested that AOA are obligate aerobes (Hatzenpichler, 2012), the presence of a phylogenetically different clade from known clusters might indicate that some novel AOA members exist and have tolerance to an extremely low DO level. The preference of AOA for low oxygen conditions has been reported in the Arabian Sea oxygen minimum zone (Pitcher et al., 2011), and a distinct cluster of AOA was also found only in the anoxic depths of the ETSP water column (Molina et al., 2010). To explore whether these distinct AOA observed in low DO conditions have ammonia oxidization activity or possess other functions as suggested recently (Weber et al., 2015) is of great value for further studies. Meanwhile, archaeal *amoA* sequences affiliated to *Nitrosopumilus* Cluster 9, 12, and 16 in this study were only found in surface sediments. The reference sequences of Cluster 9 were mainly from water column. As surface sediment contacts directly with water column, it is conceivable for aquatic microorganisms to grow in surface sediments, or the wet sediment sample may contain bottom water. The reference sequences of Cluster 12 and 16 were discovered from surface sediments of estuary and bay samples, and AOA of this group might need higher oxygen concentration to oxidize ammonia. DO in sediments of the study area ranged from ~120–250μM in the top 1 μm layer, and decreased sharply with depth (unpublished data, Dr. Guodong Song, Ocean University of China). Therefore, DO might be a significant factor affecting the niche-differentiation of AOA communities.

Variation in ammonia-oxidizing prokaryotes communities was clearer among different sites vs. the different depths at one site. The AOB community compositions of the SYS sites were more similar to each other than to the ECS sites. While SYS01 and SYS02 were dominated by *Nitrosospira* Cluster 14, ECS01, ECS02, and ECS03 were dominated by *Nitrosospira* Cluster 13. In addition, the *Nitrosomonas* lineage of AOB and the *Nitrososphaera* lineage of AOA were mainly from ECS01 and ECS02. These two sites are located near the land and have high sedimentation rates (>1 cm/y), so that some species regularly found on land can potentially be transported there along with terrestrial materials. However, species regularly found on land decreased sharply and were out-competed by marine species during transportation and long-period sedimentation, since few of them were retrieved from sediments of the other three distal sites (sedimentation rates ~0.1 cm/y). These results indicated that mud provenance and geographic location have significant impacts on the community compositions of ammonia-oxidizing prokaryotes.

In contrast to the higher diversity of AOB in mud zones of SYS, the diversity of AOB was lower than that of AOA in mud zones of ECS, consistent with the results from the Pearl Estuary (Jin et al., 2011) and SCS (Cao et al., 2011). In ECS01, located at the lower Changjiang Estuary, the diversity of AOA was high, and this may be caused by nutrients from Changjiang-diluted water and the riverine/marine water interaction. However, the diversity of AOB in this mud zone was still lower than that of SYS and decreased with depth, indicating that the diversities of AOA and AOB may be affected by different environmental factors. Statistical analysis showed that the diversity of AOA was negatively correlated with phosphate, while NO${}_{3}^{-}$, SiO${}_{3}^{2-}$, TOC, and TN were potential contributors to the diversity of AOB (Table 3). In addition, recent study found that the relative abundance of isoprenoid glycerol dialkyl glycerol tetraethers (*i*GDGTs) in the east coastal sea of China was also correlated with latitude (Lü et al., 2014). As *i*GDGTs are derived from archaeal membrane lipids, the relative abundance of *i*GDGTs may be correlated to the diversity of AOA. Previous data of AOA and AOB diversity in surface sediments of marginal seas were also collected, and compared with our results. We surprisingly found that the diversity of AOA and AOB varied in opposite trends in the shallow sediments along the western pacific shelf from north to south (Figures 2A,B). The diversity of AOA decreased at a higher latitude. The exact mechanism of this phenomenon is of great interest to explore in future studies.

### Abundance of archaeal and bacterial *amoA* at various depths and in different mud zones

AOB were numerically dominant over AOA in sediments of the mud zones in terms of *amoA* copy numbers, and the abundance of bacterial *amoA* was positively correlated with NO${}_{3}^{-}$ (Table 3). Thus, AOB might play an important role in ammonia oxidization of the studied areas. The abundance of archaeal *amoA*, but not bacterial *amoA*, showed an increasing trend with the increase of latitude in surface sediments of the western pacific marginal seas (Figure 2C). The highest abundance of archaeal and bacterial *amoA* appeared in sites ECS02 and ECS01, respectively. These two mud zones located near land can obtain sufficient nutrients for the growth of microbes from riverine inputs, waste discharges and fertilization. In addition, the higher abundance of archaeal and bacterial *amoA* in different mud zones might be due to their different responses to environmental factors. Indeed, a preference for low NH${}_{4}^{+}$ level has been reported for cultured AOA (Hatzenpichler et al., 2008; Lehtovirta-Morley et al., 2011), and a higher tolerance of AOB to NH${}_{4}^{+}$ (50–1000mM) than that of AOA (10–20 mM) has been demonstrated in cultured AOA and AOB (Hatzenpichler, 2012). In surface sediments of the East China Sea, it has been reported that β-AOB dominated the area of high ammonium level while AOA preferred high salinity area (He et al., 2015). In addition, the abundance of archaeal *amoA* was positively correlated with SiO${}_{3}^{2-}$ while bacterial *amoA* was negatively correlated (Table 3). The concentration of NH${}_{4}^{+}$ in sediments of ECS01 was much higher than in ECS02, while the concentration of the sedimentary SiO${}_{3}^{2-}$ and the bottom water salinity had an opposite trend (Table S1). These trends can explain the differentiation in abundance of archaeal and bacterial *amoA* in ECS01 and ECS02.

Few studies have focused on vertical distributions of AOA and AOB in marine sediments. Across samples of all depths (0–33 cm) in this study, the average abundance of archaeal and bacterial *amoA* in the upper layers (< 5 cm) was greater than that in the deeper layers. The abundance of archaeal *amoA* showed a slighter decrease with the increase of depth than that of bacterial *amoA* in this study and archaeal *amoA* in the East Sea of Korea (Park et al., 2008). Similar to the result reported in a sandy ecosystem (Leininger et al., 2006), the ratio of archaeal and bacterial *amoA* in this study increased with depth. This might be caused by DO gradients as low oxygen might select for the numerical dominance of AOA and the coexistence of AOA and AOB (Lam et al., 2007; Santoro et al., 2008). However, the exact mechanism need to be determined in future studies.

### Response of ammonia-oxidizing prokaryotes community to environmental factors

Variation in AOA community was significantly correlated with δ^15^N_TN_, SiO${}_{3}^{2-}$, and NH${}_{4}^{+}$ (Figure 6A), whereas that in AOB community showed significant correlation with NO${}_{3}^{-}$ and δ^13^C (Figure 6B). While both NH${}_{4}^{+}$ (the substrate of ammonia mono-oxygenase) and NO${}_{3}^{-}$ (the ultimate product of nitrification) have previously been identified as major environmental factors influencing the community compositions of AOA or AOB (Shen et al., 2008; Wankel et al., 2011; Zheng et al., 2014; Chen et al., 2014b), our discovery that δ^15^N_TN_ and δ^13^C were correlated with the community composition and distribution of AOA and AOB with the *amoA* gene is without precedent. This observation might be explained by stable isotope fractionation in AOA and AOB. Different N isotopic discriminations for ammonia oxidation have been found in different pure-cultured AOB strains, as well as enrichments in pelagic AOA. The ε_*AMO*_ of five different pure-cultured AOB strains ranged from 14.2 to 38.2%0in one study (Casciotti et al., 2003), and the ^15^ε_*NH*3_ of three enrichments of AOA ranged from 13 to 41%0in another (Santoro and Casciotti, 2011). These discriminations lead to different isotopic compositions in produced NO${}_{2}^{-}$; after converstion to N_2_ and N_2_O during denitrification, these gases are eventually released into the atmosphere. Over time, the concentration of δ^15^N_TN_ in sediments with different AOA and AOB communities would therefore be exptected to change.

SiO${}_{3}^{2-}$ was found to be a very important environmental factor for the relative abundance and community composition of archaeal and bacterial *amoA* in this study (Table 3; Figure 6A). Zhang et al. (2014) also found that SiO${}_{3}^{2-}$ was a significant variable explaining the clustering pattern of free-living AOA and AOB communities in water column sampled in the Changjiang Estuary. In the present study, correlations between the concentration of SiO${}_{3}^{2-}$ and the abundance of archaeal and bacterial *amoA* were opposite (negatively for AOB and positively for AOA). The phenomenon became more evident when the abundance of archaeal *amoA* in the top five sediment layers was taken into account; the abundance was significantly correlated with the concentration of SiO${}_{3}^{2-}$ (*P* = 0.0023). This may be attributed to microorganisms which mediate dissolution of siliceous deposits (Bidle and Azam, 1999). These microorganisms might consume NH${}_{4}^{+}$, as a negative correlation was found between concentrations of NH${}_{4}^{+}$ and SiO${}_{3}^{2-}$ (*P* = 0.031) in this study. As cultured AOA are adapted to considerably lower NH${}_{4}^{+}$ concentration than AOB species (Schleper, 2010), AOA might have more competitive advantage than AOB in uptake of NH${}_{4}^{+}$ when concentration of NH${}_{4}^{+}$ was low. The possibility that SiO${}_{3}^{2-}$ might be essential for the growth of AOA could not be excluded since Si is involved in the cellular structure of some microorganisms (Chen et al., 2014a).

In conclusion, this study showed that the community composition and abundance of ammonia-oxidizing prokaryotes varied greatly in different mud zones and at different depths of ECMS. The mechanism governing the distribution of ammonia-oxidizing prokaryotes needs to be further elucidated.

## Author contributions

SY carried out the laboratory work, data analysis, and drafted the manuscript. PY processed the raw data and drafted the manuscript. JL and BZ helped collect sediment samples and qPCR analysis. GZ, MZ, and ZY participated in the design of the study and helped to draft the manuscript. XZ conceived the study, and revised and finalized the manuscript. All authors read and approved the final manuscript.

## Funding

This work was supported by the National Natural Science Foundation of China through grants 41476112, 41276141, 41521064, and 41376088.

### Conflict of interest statement

The authors declare that the research was conducted in the absence of any commercial or financial relationships that could be construed as a potential conflict of interest.
