# Supplementary material for: Diversity, Abundance, and Niche Differentiation of Ammonia-Oxidizing Prokaryotes in Mud Deposits of the Eastern China Marginal Seas
Source: Front Microbiol. 2016 Feb 12;7:137. doi: 10.3389/fmicb.2016.00137 (PMC4751261; doi:10.3389/fmicb.2016.00137)
Supplement: Supplementary file 1 [file DataSheet1.DOCX]

**Supplementary Material for Yu et al.**

**Supplementary Table, Figures and Figure legends**

**Table S1.** Sampling location, bulk sedimentary OC parameters, and dissolved inorganic nutrients in pore waters of the five sediment cores sampled in 2013 from the eastern China marginal seas

| Station | Longitude (°E) | Latitude  (°N) | %TOC | %TN | C/N  (mol/mol) | δ^13^C(‰) | δ^15^N_TN_(‰) | NO_3_^-^  (μmol L^-1^) | NO_2_^-^  (μmol L^-1^) | NH_4_^+^  (μmol L^-1^) | PO_4_^3-^  (μmol L^-1^) | SiO_3_^2-^  (μmol L^-1^) | Salinity |
| --- | --- | --- | --- | --- | --- | --- | --- | --- | --- | --- | --- | --- | --- |
| **SYS01** | 123.50 | 35.00 | 0.90±0.07 | 0.13±0.01 | 8.15±0.41 | -22.11±-0.18 | 5.32±0.25 | 1.23±0.99 | 0.27±0.15 | 56.5±13.6 | 7.41±1.77 | 235.8±28.5 | 30.31 |
| **SYS02** | 123.96 | 34.44 | 0.77±0.08 | 0.12±0.02 | 7.78±1.05 | -21.96±-0.18 | 4.22±0.43 | 1.21±0.81 | 0.28±0.13 | 70.4±35.5 | 7.88±1.92 | 265.1±32.8 | 31.15 |
| **ECS01** | 122.73 | 31.02 | 0.63±0.05 | 0.11±0.02 | 7.12±0.90 | -22.75±-0.18 | 2.84±0.59 | 0.32±0.65 | 0.17±0.05 | 285.9±182.4 | 2.55±2.45 | 284.4±119.7 | 33.38 |
| **ECS02** | 122.58 | 28.73 | 0.79±0.05 | 0.13±0.01 | 7.26±0.52 | -21.66±-0.18 | 3.91±0.20 | 0.60±0.37 | 0.29±0.14 | 33.2±19.7 | 7.69±5.55 | 399.0±36.8 | 34.42 |
| **ECS03** | 125.50 | 31.99 | 0.55±0.06 | 0.09±0.01 | 7.35±0.29 | -21.61±-0.07 | 5.50±0.25 | ND | ND | ND | ND | ND | 32.34 |

ND: not detected.

**

**

**Fig. S1** Organic parameters of sediments and inorganic nutrients in sediment pore waters from the mud deposits at eight depths in the China Eastern Marginal Seas. **a**. Organic parameters. **a1**, total organic carbon (TOC); **a2**, total nitrogen (TN); **a3**, C/N ratios; **a4**,stable carbon isotopes (δ13C); **a5**, nitrogen isotopes (δ15N). **b**. Inorganic nutrients. **b1**, NO_3_^-^; **b2**, NO_2_^-^; **b3**, NH_4_^+^; **b4**, PO_4_^3-^; **b5**, SO_3_^2-^.


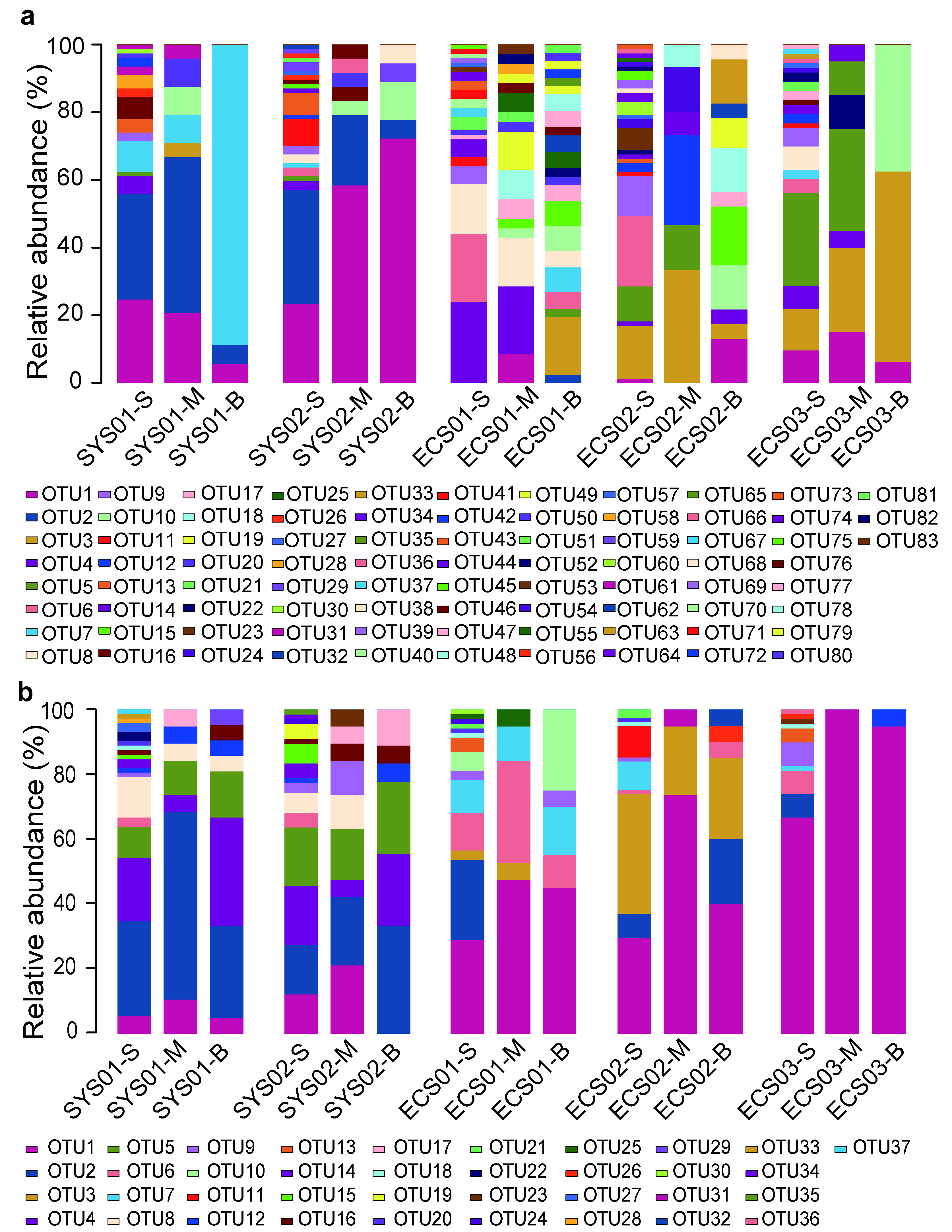


**Fig. S2** Distribution and relative abundance of OTUs of AOA and AOB in the mud deposits of the China Eastern Marginal Seas. **A**, AOA; **b**, AOB.


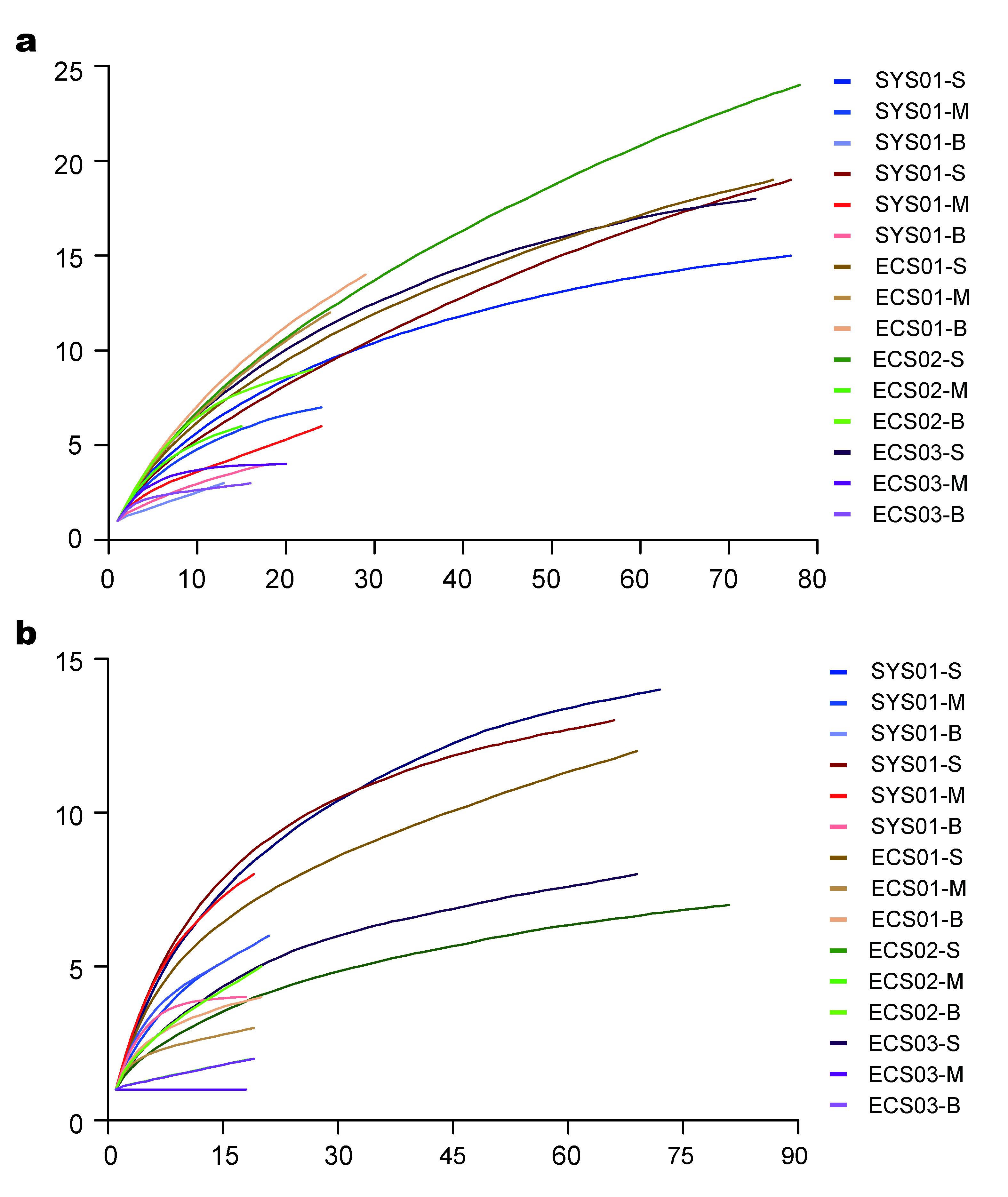


**Fig. S3** Rarefaction curves of the OTUs in the amoA gene clone libraries constructed with the sediment samples collected from the mud deposits of the China Eastern Marginal Seas. **a**, AOA; **b**, AOB.





**Fig. S4** Distance-based neighbor-joining phylogenetic tree of the bacterial *amoA* sequences recovered from mud deposits of the eastern China marginal seas and their closest matches in GenBank. Bootstrap support values > 50% (1000 replicates) are shown.

**
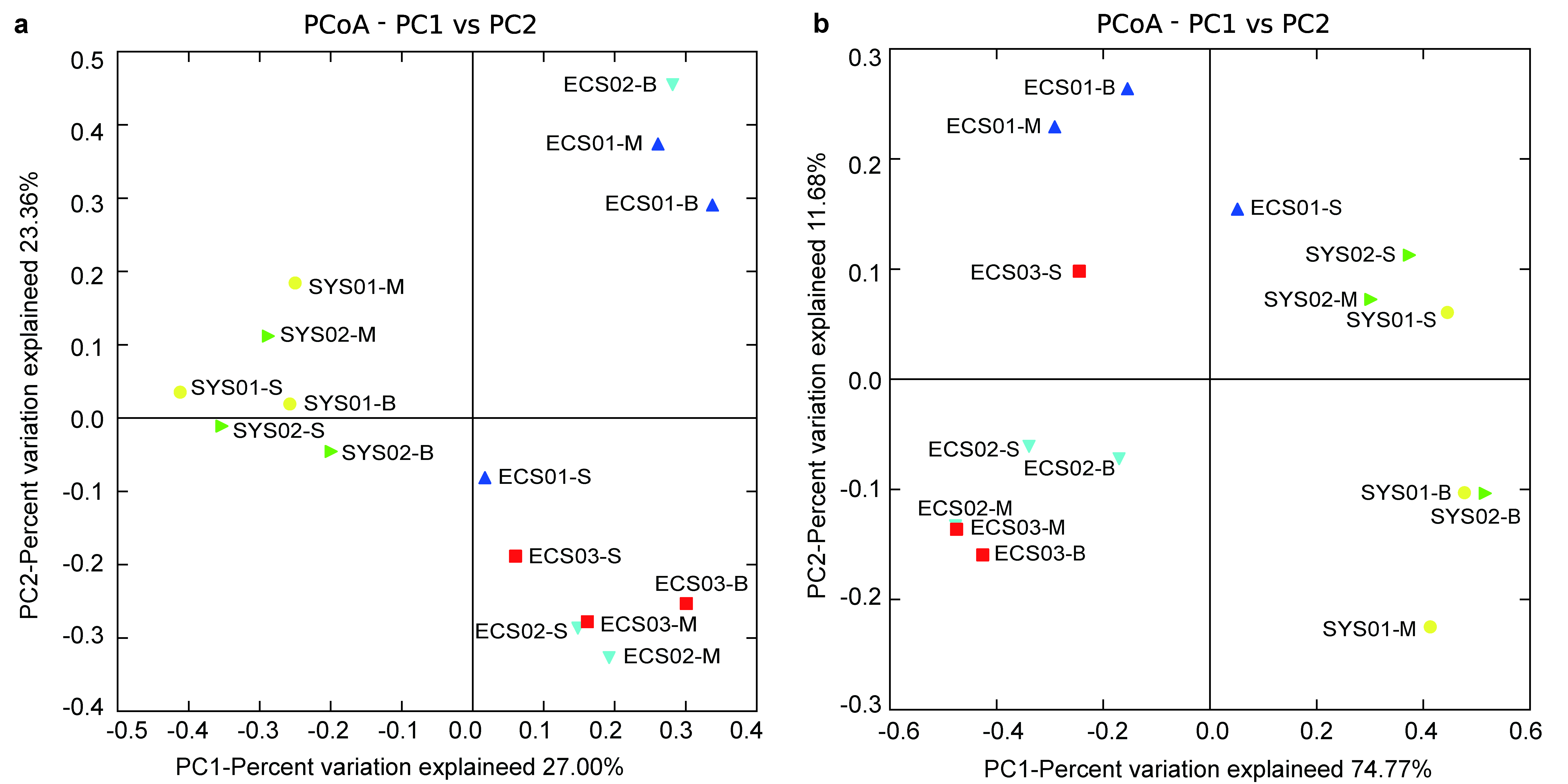
**

**Fig. S5** Ordination diagrams of the archaeal and bacterial *amoA* assemblages from mud deposits of the eastern China marginal seas calculated with weighted UniFrac PCoA analysis. The plot of the first two principal-coordinate axes (P1 and P2) for PCoA and the distributions of the AOA (**a**) and AOB (**b**) *amoA* assemblages (designated by the sampling station names) in response to these axes are shown.
